# Variation in the Phenolic Profile and Antioxidant, Antihyperglycemic, and Anti-Inflammatory Activity in Leaves of *Cotoneaster zabelii* during Growing Season

**DOI:** 10.3390/molecules29194745

**Published:** 2024-10-08

**Authors:** Agnieszka Kicel, Anna Magiera, Monika Anna Olszewska

**Affiliations:** Department of Pharmacognosy, Faculty of Pharmacy, Medical University of Lodz, 1 Muszynskiego St., 90-151 Lodz, Poland; anna.magiera@umed.lodz.pl (A.M.); monika.olszewska@umed.lodz.pl (M.A.O.)

**Keywords:** *Cotoneaster*, seasonal variation, phenolic composition, antioxidant, antihyperglycemic, anti-inflammatory activity

## Abstract

*Cotoneaster zabelii* is a medicinal plant that is beneficial due to its polyphenol-rich leaves. In the course of optimizing the harvest time for *C. zabelii* cultivated in Poland, the leaf samples were collected monthly during the annual plant vegetation season, and the hydromethanolic leaf extracts were evaluated for their phenolic composition and model biological activities, including antioxidant, antihyperglycemic, and anti-inflammatory effects in vitro. The phenolic profiles were analyzed using UHPLC-PDA-ESI-MS^3^, HPLC-PDA, and spectrophotometric methods (total phenolic content, TPC) to understand their seasonal variability and its correlation with bioactive properties. The identified phenolic compounds included caffeic acid derivatives, flavan-3-ols (especially (−)-epicatechin and procyanidins B-type), and flavonoids like quercetin mono- and diglycosides. Leaves harvested in July and October contained the highest polyphenolic levels and demonstrated significant antioxidant activity in most tests. The leaves harvested in July, September, and October showed optimal anti-inflammatory effects, whereas the highest antihyperglycemic activity was observed in the leaves collected from June to July. Regarding polyphenolic levels and bioactivity, the summer and autumn months appear to be the most advantageous for harvesting leaf material of optimal quality for phytotherapy.

## 1. Introduction

Phenolic compounds are widely investigated in various plant species due to their high diversity and wide-ranging biological effects, frequently used in traditional medicine, phytotherapy, and functional food applications. However, the presence and abundance of these plant compounds in a collected plant material are strongly influenced by numerous parameters, primarily the type of plant tissue analyzed, the physiological (growth) age of the plant, its different stages of development related to the date of harvest, environmental conditions, cultivation practices, and post-harvest conditions, including the storage parameters of the raw material [1]. The seasonal variability in the quality and quantity of these plant metabolites modifies the biological activity of plant extracts. Consequently, samples of the same plant collected at different points of the growing season usually differ significantly in polyphenolic content, which affects their pharmacological properties successively manifested throughout the year [2].

The presence of polyphenols in the leaves and fruits of *Cotoneaster* species is referred to by several authors, who suggest the pivotal role of these compounds in the pharmaceutical properties associated with these plants [3,4,5,6]. *Cotoneaster zabelii* C.K. Schneid is a rosaceous species native to China and naturalized in Europe, where it is extensively cultivated as an ornamental plant. With other *Cotoneaster* taxa, it is also considered a valuable source of traditional medicines, exhibiting cardiotonic, diuretic, expectorant, antiviral, antidiabetic, and antispasmodic properties [7]. As indicated by the previous LC-MS studies of the *C. zabelii* leaves and fruits, most of these bioactivities might be attributed to low-molecular weight polyphenols (over thirty analytes detected), especially B-type proanthocyanidins, flavonoids (mainly quercetin glycosides), and caffeoylquinic and caffeoylmalic acids [5,6,8]. The hydroalcoholic extracts from the leaves also showed high total antioxidant capacity, potent anti-inflammatory properties, and significant ability to inhibit diabetes-related digestive enzymes and nonenzymatic protein glycation [5,9,10].

Although the polyphenolic composition of *C. zabelii* extracts is relatively well recognized, there are currently no experimental data available on how it varies with the seasons, nor on how these variations relate to the biological properties of the extracts and their therapeutic potential. Therefore, it is essential from an economic and industrial point of view to explore the topic in a systematic variability study of *C. zabelii* leaves throughout the growing season. Analyzing the polyphenolic profile and in vitro antioxidant, antidiabetic, and anti-inflammatory activity in correlation with the stage of leaf maturity is required for selecting an optimal harvesting time to receive plant material of the quality adequate for medicinal application. Therefore, this work aimed to evaluate changes in the polyphenolic composition, along with antioxidant activity and inhibitory effect on the digestive and pro-inflammatory enzymes, as well as the relationships between the tested parameters in monthly harvested leaf samples of *C. zabelii* during cultivation in Poland.

## 2. Results

### 2.1. Seasonal Variability in the Levels of Polyphenolic Components

**Figure 1:**
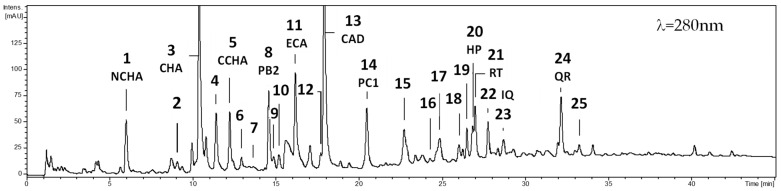
The representative UHPLC-UV chromatogram recorded at 280 nm for the methanol–water (7:3, *v*/*v*) extract of *C. zabelii* leaves collected in July (VII) 2020. The identified polyphenolic components: 1, 3-O-caffeoylquinic acid, NCHA; 2, caffeic acid derivative; 3, 5-*O*-caffeoylquinic acid, CHA; 4, dicaffeoylquinic acid isomer; 5, 4-*O*-caffeoylquinic acid, CCHA; 6, dicaffeoylquinic acid isomer; 7, procyanidin dimer B-type; 8, procyanidin B2 PB2; 9, procyanidin trimer B-type; 10, 5-*p*-coumaroylquinic acid; 11, (−)-epicatechin, ECA; 12, procyanidin tetramer B-type; 13, caffeoylmalic acid, CAD; 14, procyanidin C1 PC1; 15, procyanidin tetramer B-type; 16, procyanidin tetramer B-type; 17, procyanidin dimer hexoside; 18, quercetin-rhamnoside-hexoside; 19, quercetin dirhamnoside; 20, hyperoside HP; 21, rutin, RT; 22, isoquercitrin, IQ; 23, procyanidin dimer B-type; 24, quercitrin, QR; 25, quercetin hexoside derivative.

The qualitative analysis of hydromethanolic extracts from the leaves of *C. zabelii* using UHPLC-PDA-ESI-MS^3^ confirmed the presence of 25 phenolic compounds (Figure 1). These constituents included phenolic acids, mainly caffeic acid derivatives; flavan-3-ols, primarily (−)-epicatechin and procyanidins B-type; and flavonoids like quercetin mono- and diglycosides. As previously described, these compounds were identified based on their chromatographic and spectral characteristics [5,8]. While a direct comparison of UHPLC phenolic profiles of leaf samples collected in different months did not reveal any qualitative variations, it indicated notable quantitative variability for most of the polyphenolic groups and individual compounds.

**Figure 2:**
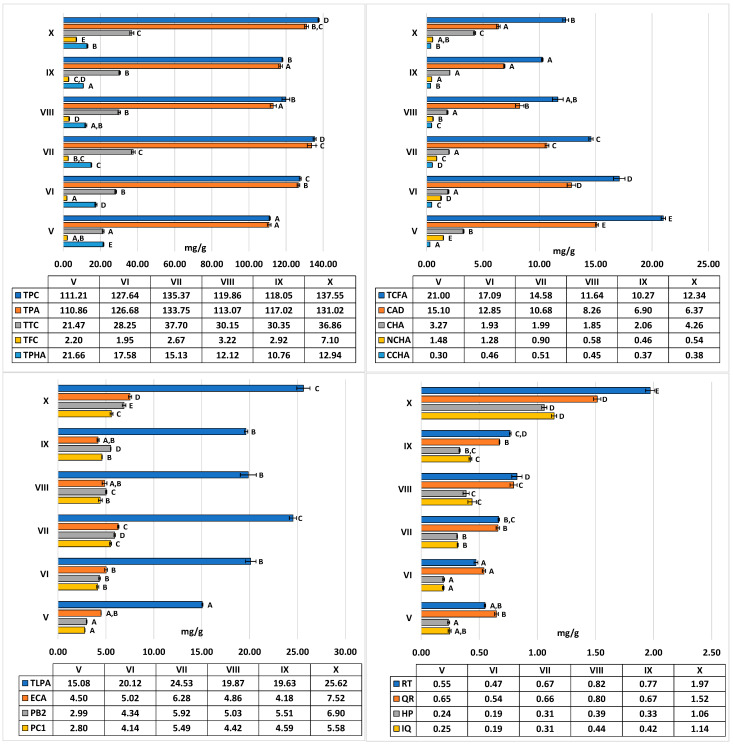
Seasonal fluctuations from May (V) to October (X) 2020 in the groups and individual polyphenols in the *C. zabelii* leaves. Data reported as the mean values ± standard error SE (*n* = 3) based on the dry weight of the plant material. For each graph, the different capital letters A–E denote statistically significant differences (*p* < 0.05). Parameters: TPC, total phenolic content determined by the Folin–Ciocalteu method and expressed in gallic acid equivalents (GAE); TPA, total proanthocyanidin content performed by the n-butanol/HCl method and expressed in cyanidin equivalents (CYE); TTC, total tannin content determined by the hide powder test and expressed in pyrogallol equivalents (PRE); TFC, the sum of individual flavonoids (HPLC-PDA); TCFA, the sum of caffeic acid derivatives (HPLC-PDA); TPHA, the sum of individual caffeic acid derivatives (TCFA) and *p*-coumaroyloquinic acids (CPCA) (HPLC-PDA); CAD, caffeoylmalic acid; CHA, NCHA, and CCHA, 5-*O*-, 3-*O*-, and 4-*O*-caffeoylquinic acids, respectively; TLPA, the sum of individual low-molecular-weight proanthocyanidins (HPLC-PDA); ECA, (−)-epicatechin; PB2, procyanidin B2; PC1, procyanidin C1; RT, rutin; QR, quercitrin; HP, hyperoside; IQ, isoquercitrin.

**Figure 3:**
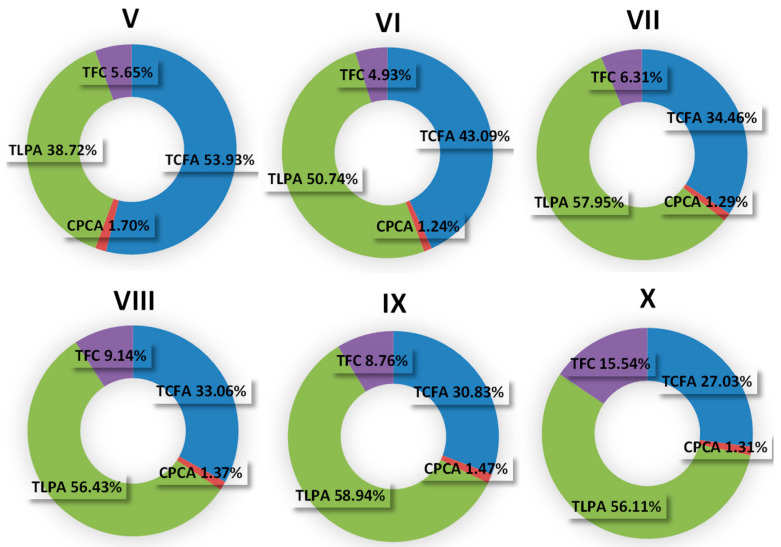
Contribution of individual groups of compounds to total phenolic contents determined by HPLC-PDA in *C. zabelii* leaves collected from May (V) to October (X) 2020; TFC, the sum of individual flavonoids; TCFA, the sum of caffeic acid derivatives; CPCA, the sum of *p*-coumaroyloquinic acids; TLPA, the sum of individual low-molecular weight proanthocyanidins.

Throughout the one-year growing season, the leaves of *C. zabelii* revealed subtle yet statistically significant (*p* < 0.05) variations in the total phenolic content (TPC), quantified in terms of gallic acid equivalents (GAE). The highest levels were observed in July and October, while the lowest were noted in May and September (Figure 2). Variations in the TPC levels (111.2–135.4 mg GAE/g dry leaf mass) ranged up to 19.2% of the highest value (Table 1). The calculated coefficient of variation (CV) was 8.3% (Table 1). In the content of individual groups of analytes (Figure 2 and Figure 3), diverse variations were observed, ranging from 17.1% to 72.5% of maximum levels for proanthocyanidins, tannins, flavonoids, and phenolic acids. The CV values for these groups remained within the range of 7.9% to 56.8% (Table 1). Furthermore, the total levels of proanthocyanidins (low-molecular weight—TLPA; and total—TPA) and tannins (TTC) exhibited seasonal dynamics similar to TPC, indicating that their highest concentrations were observed during summer and autumn. The peak levels of these metabolites were reached in July and October before the fall of autumn leaves, suggesting an accelerated synthesis of both high-molecular weight proanthocyanidins, low-molecular weight oligomers, and monomeric flavan-3-ols. This trend is also reflected in the seasonal dynamics of the individual compound variability observed within this group. The highest concentrations of (−)-epicatechin (ECA) and procyanidins B2 and C1 (PB2 and PC1) were found in July and October. The increase in proanthocyanidin levels during autumn was also accompanied by the increase in the flavonoid content. All compounds of this group, primarily RT, QR, HP, and IQ, exhibited a similar variability trend, with the highest contents noted for October leaves. Different seasonal dynamics were observed for caffeic acid derivatives, showing at least two distinct accumulation profiles for compounds of this group. The first was typical of caffeoylmalic acid (CAD), which reached its maximum level in spring, as early as May, with a decreasing content until autumn. On the other hand, the content of chlorogenic acid (CHA) sharply declined one month after reaching its highest level in May, with relatively little change during summer, before rising again to the maximum in October. However, the cumulative contribution of these two dominant compounds in TPHA remained stable throughout the season, falling within a narrow range of 82.1–84.8%

### 2.2. Seasonal Variability in the Antioxidant, Antihyperglycemic, and Anti-Inflammatory Activity

The observed variations in the polyphenolic profiles were likely to affect the biological potential of *C. zabelii* leaves harvested at different stages of their annual development. Indeed, seasonal fluctuations in the antioxidant, antihyperglycemic, and anti-inflammatory activities were demonstrated, with the highest capacities observed, depending on the in vitro model, for leaves collected in July and October (DPPH and FRAP tests), in June and July (O_2_^•−^ -scavenging and α-glucosidase inhibition), and in July, September, and October (hyaluronidase inhibition) (Table 2). Moreover, positive correlations (*p* < 0.05) were revealed between antioxidant activity and phytochemical parameters, primarily the levels of TPC, TPA (for the DPPH and FRAP tests), and TTC and TLPA (for the DPPH test only) (Table 3). Regarding the antihyperglycemic activity, a statistically significant (*p* < 0.05) correlation was found with the TPA level in the α-glucosidase inhibition test, while for the anti-inflammatory activity, it was found with the levels of TTC, TLPA, and TPHA in a hyaluronidase inhibition test. Among the identified compounds, derivatives of flavan-3-ol, from monomers to polymers, were mainly responsible for the observed positive correlations with the tested activities. For instance, the elevated antioxidant potential of the leaves, particularly observed during the summer and autumn months, was related to the elevated content of both low- and high-molecular weight proanthocyanidins. Similarly, both low- and high-molecular weight proanthocyanidins were identified as the primary contributors to the anti-inflammatory activity (ability to inhibit hyaluronidase) of the studied leaf samples. In the case of α-glucosidase inhibition (antihyperglycemic action), the most active compounds were high-molecular weight proanthocyanidins, predominantly accumulated in leaves from the summer period. An interesting observation is that the CV values for activity parameters (8.3–25.8%) were generally lower or similar to those observed for the TPC, TPA, and TLPA levels (Table 1 and Table 2). On the one hand, this fact further supports the conclusion of the essential role of proanthocyanidins in responding to the environmental changes during the foliar development of *C. zabelii* leaves and, on the other hand, it suggests that the vital synergic effect of various polyphenols might arise and lower the variability of their total impacts.

## 3. Discussion

Polyphenolic compounds, representing structures ranging from simple phenolic acids to polymeric tannins, are integral components of numerous plants. They play a significant role in neutralizing the effects of prolonged oxidative stress and inflammation and chiefly contribute to the preventive potential of herbal medicines against chronic civilization diseases, such as cardiovascular diseases, neurodegenerative disorders, diabetes, and accelerated aging [11]. Seasonal dynamics of polyphenol biosynthesis thus emerge as a crucial factor in producing plant-derived medicinal products, influencing their pharmacological effectiveness and economic value. Hence, the seasonal variations in polyphenolic profiles and their associated activities, including antioxidant and anti-inflammatory properties, are commonly addressed in optimizing plant sources for medicinal application. Such interrelationships were observed, e.g., in the genus *Crataegus* (Rosaceae), whose representatives are renowned for well-documented beneficial health effects. For instance, the leaves of *C. pentagyna* have been found to accumulate significantly higher levels of polyphenols when harvested in September than in other parts of the growing season [12]. Similarly, the leaves of *C. pinnatifida* contained elevated levels of polyphenols when collected during the late summer and autumn months [13,14]. Following the literature data, the seasonal variations observed in the present study for the *C. zabelii* leaves align with the general trend, indicating that summer and autumn are the most advantageous for collecting plant material for functional application. However, it is noteworthy that, opposite to the above-mentioned hawthorn leaves, the investigated *C. zabelii* leaves exhibited relatively moderate seasonal fluctuations for both polyphenol levels and antioxidant, antihyperglycemic, and anti-inflammatory properties of the leaf extracts. Therefore, the studied species may be profitable for industrial plant production.

Phytochemical investigations of polyphenols in *C. zabelii* conducted in the current and prior studies [5,8] have confirmed the phytochemical profile as potentially beneficial to human health due to the presence of a rich complex of bioactive proanthocyanidins, phenolic acids, and flavonoids. This study reports for the first time that all of these groups of polyphenols are prone to quantitative changes during the growing season, and these variations contribute to the changing of the pharmacological value of the leaf material and extracts, with the most influential effects occurring from proanthocyanidins on the plant activity. Moreover, a vital synergic effect of various polyphenols was observed in *C. zabelii* leaves that might explain lower or similar CV values noted for the activity parameters compared with the phenolic levels. These results confirm the previously postulated hypothesis [15,16,17] that proanthocyanidins of various levels of polymerization, exerting their effects through synergistic action with other polyphenols, are essential components impacting the antioxidant, antihyperglycemic, and anti-inflammatory capacity of leaves of various plants during their annual development.

Low-molecular weight proanthocyanidins (oligomers and polymers of (−)-epicatechin and (+)-catechin), forming a substantial fraction of the analyzed *C. zabelii* leaves and positively correlated with their antioxidant and anti-inflammatory activity, are widely recognized for their protective effects in cardiovascular diseases [18,19]. Proanthocyanidins downregulate the pro-inflammatory pathways and inhibit the release of pro-inflammatory cytokines and transcription factors, helping to mitigate vascular inflammation, oxidative stress, and atherosclerosis. Moreover, they counteract the endothelial dysfunctions, stabilize the atherogenic plaque, and increase the bioavailability of nitric oxide (NO), thus contributing to the normalization of blood pressure and lowering cardiovascular risk [18,19]. The high content of proanthocyanidins in *C. zabelii* leaves and their primary impact on the leaf extract response in the screening panel of free radical scavenging, FRAP, and hyaluronidase inhibition in vitro tests, is thus in accordance with the traditional application of the plant material in cardiovascular disorders [7,8].

The accumulated research also indicates that proanthocyanidins are one of the primary metabolites aiding the plant adaptation to changing environmental conditions and chiefly contributing to the seasonal phytochemical variability of many plants. Proanthocyanidins form an essential part of the antioxidant defense system, protecting aerial plant parts, primarily leaves and flowers, from oxidative damage by neutralizing free radicals. This protection is crucial in seasons with elevated environmental stress, such as high sunlight intensity or drought. Therefore, the concentration of proanthocyanidins in plant leaves can vary within various seasons, often increasing during periods when the plant is more likely to encounter stress, such as summer or early fall [20,21,22], which was also observed for *C. zabelli* leaves in the present study.

Caffeic acid derivatives, identified in the current research as the predominant fraction of phenolic acids in *C. zabelii* leaves, are also known as potent antioxidants and anti-inflammatory agents with favorable bioavailability in humans after oral administration. Numerous studies have demonstrated the protective effects of chlorogenic acid against cardiovascular pathologies, such as endothelial dysfunction, vascular hypertrophy, and hypertension. Moreover, this compound is considered an effective anti-atherogenic agent, able to reduce hypercholesterolemia risk factors, including total cholesterol, triglycerides, LDL, and LDL/HDL ratio in vivo [23,24].

Caffeic acid derivatives also contribute to seasonal plant variability through their role in defense mechanisms. Chlorogenic acid isomers act as antioxidants, helping plants manage oxidative stress caused by environmental factors, including high light intensity, UV radiation, and temperature fluctuations. During specific parts of growing seasons, particularly early summer, when these stress conditions are more intense, plants may increase the production of chlorogenic acid for tissue protection. Furthermore, chlorogenic acid contributes to the plant’s defense against herbivores and pathogens. Its levels in plant leaves may rise when these threats are more prevalent, which often corresponds to specific seasons when insects or microbial activity is elevated. Chlorogenic acid biosynthesis is influenced by light and temperature, both of which vary seasonally. For example, longer daylight hours and higher summer temperatures can stimulate chlorogenic acid production, while shorter days and cooler temperatures in autumn may lead to a decrease [25,26]. All of these data follow and explain our observation of an increased content of caffeic acid derivatives in *C. zabelii* leaves between May and July.

Flavonoids, including quercetin glycosides, as another group of *C. zabelii* leaf polyphenols, also offer a wide range of health benefits due to their antioxidant, anti-inflammatory, vasoprotective, vasodilating, anti-cancer, immune-supporting, neuroprotective, and metabolic effects [27,28,29]. In general, flavonoids are especially prone to seasonal changes among various phenolics and are dynamically biosynthesized in plants in response to changing environmental factors. Increased sunlight during spring and summer typically boosts the production of quercetin glycosides due to the urgent need for UV protection. In autumn, when the leaves are prepared for senescence and falling, there may be an increase in the levels of certain flavonoids observed, including quercetin glycosides, which contributes to the color changes in autumn leaves (e.g., yellowing due to flavonoid accumulation) and is partly associated with the decrease in the dry mass of leaves [30,31]. Indeed, for *C. zabelii* leaves, we observed the highest flavonoid content in autumn, with a sharp increase to the peak level in October at the end of the vegetation season.

In light of the revealed compositional changes of polyphenolic components and their antioxidant and anti-inflammatory activities and antidiabetic effects in vitro, it can be concluded that proanthocyanidins, caffeic acid derivatives, and flavonoids are synergistically responsible for the observed activity of the *C. zabelii* leaves; however, the primary impact comes from proanthocyanidins. Our observations form the basis for selecting the most promising harvesting period for collecting plant material for medicinal purposes. Moreover, the study shows the high predictive value of the phytochemical profiles of *C. zabelii* leaves relative to their biological potential in oxidative stress, inflammation, and diabetes-related disorders, indicating their utility in the quality control of plant material.

Evaluating the plant material quality and phytochemical consistency is essential for ensuring the plant’s pharmacological effectiveness. Consequently, quality control is strongly recommended even before harvesting to confirm the presence of biologically active compounds at required levels. Understanding the seasonal variability in polyphenolic content and biological activity is particularly important for plant materials available throughout the whole growing season, primarily leaves. Therefore, the present study provides an effective analytical tool for such investigations and control procedures in *Cotoneaster* plants.

## 4. Materials and Methods

### 4.1. Chemical and Reagents

HPLC-grade purity reagents and standards such as 2,2-diphenyl-1-picrylhydrazyl (DPPH), 2,4,6-tris- (2-pyridyl)-s-triazine (TPTZ), α-glucosidase from *Saccharomyces cerevisiae*, *p*-nitrophenyl-α-D-glucopyranoside (PNPG), bovine testis hyaluronidase, xanthine oxidase from bovine milk, xanthine, nitrotetrazolium blue chloride, chlorogenic acid hemihydrate (5-*O*-caffeoylquinic acid), 3-*O*- and 4-*O*-caffeoylquinic acids, hyperoside semihydrate, isoquercitrin, and rutin trihydrate were obtained from Sigma-Aldrich (St. Louis, MO, USA); meanwhile, (−)-epicatechin and procyanidins B-2 and C-1 were obtained from PhytoLab GmbH (Vestenbergsgreuth, Germany). Quercitrin and caffeoylmalic acid standards have been previously isolated in our laboratory from *C. zabelii* leaves [9]. The HPLC-grade solvents, acetonitrile (ACN), and orthophosphoric acid (H_3_PO_4_) used for LC analyses were from Avantor Performance Materials (Gliwice, Poland). For spectrophotometric tests, the absorbance was measured using a UV-1601 Rayleigh spectrophotometer (Beijing, China) or a SPECTROStar Nano microplate reader (BMG Labtech, Ortenberg, Germany). The samples were incubated at a constant temperature using a BD 23 incubator (Binder, Tuttlingen, Germany) for activity tests. The UHPLC-PDA-ESI-MS^3^ and HPLC-PDA studies were performed using previously described equipment [6,9].

### 4.2. Plant Material and Sample Preparation

Leaves of *Cotoneaster zabelii* C.K. Schneid (Rosaceae) were collected throughout a one-year growing season in 2020, from May to October, at the coordinates 51°45′ N, 19°24′ E, in the Botanical Garden of Lodz. Plant material samples (approximately 10 g) were collected each month from a single, consistently selected shrub. The authentication of the species was performed by the authors and plant taxonomists of the Garden. Voucher specimens were archived in the herbarium of the Department of Pharmacognosy, Medical University of Lodz (Poland), under following reference numbers: KFG/05.20/CZBL, KFG/06.20/CZBL, KFG/07.20/CZBL, KFG/08.20/CZBL, KFG/09.20/ CZBL, and KFG/10.20/CZBL.

The leaf extracts were prepared using a previously outlined method [5] involving triple reflux extraction with methanol–water (7:3, *v*/*v*). For the UHPLC-MS qualitative analysis, the extracts from each month, obtained from 1 g of plant material, were independently combined, evaporated to dryness under vacuum, and lyophilized (extraction yield range of 29.9–33.3%). The extract samples (15 mg) were dissolved in 3 mL of methanol–water (7:3, *v*/*v*). For the HPLC-PDA quantitative study and activity tests, the crude extracts obtained from 100 or 500 mg of plant material were diluted with the same solvent to 100 mL.

### 4.3. Phytochemical Profiling

The metabolite profiling and quantitative study of individual phenolic compounds were performed by UHPLC-PDA-ESI-MS^3^ and HPLC-PDA analyses using the same equipment and procedures as described previously [6,9].

The UHPLC-PDA-ESI-MS^3^ qualitative analyses were conducted using a Kinetex XB-C18 column (150 × 2.1 mm, 1.7 μm; Phenomenex Inc., Torrance, CA, USA). The gradient solvent system comprised two components: water–formic acid (100:0.1, *v*/*v*) as solvent A and acetonitrile–formic acid (100:0.1, *v*/*v*) as solvent B. The elution profile was as follows: 0–45 min, 6–26% (*v*/*v*) B; 45–55 min, 26–95% B; 55–60 min, 95% B; and 60–63 min, 95–6% B (equilibration). The flow rate was set at 0.3 mL/min, the column temperature was maintained at 25 °C, and the injection volume was 3 µL. The UV-Vis spectra were recorded in the 200–600 nm range, and chromatograms were acquired at 280, 325, and 350 nm wavelengths. The LC eluate was directly introduced into the ESI interface without splitting and was analyzed in a negative ion mode. The scan ranged from m/z 70 to 2200. The MS^2^ and MS^3^ fragmentations were obtained using the auto MS/MS mode for the most abundant ions at the analysis time. The instrument settings were as follows: nebulizer pressure: 40 psi; dry gas flow: 9 L/min; dry gas temperature: 300 °C; and capillary voltage: 4.5 kV.

The quantitative HPLC-PDA analyses were conducted using the C18 Ascentis^®^ Express column (2.7 mm, 75 × 4.6 mm i.d.; Supelco, Bellefonte, PA, USA). The gradient solvent system consisted of water–orthophosphoric acid (99.5:0.5, *v*/*w*) as solvent A and acetonitrile as solvent B. The elution profile was as follows: 0–14 min, 6–30% B (*v*/*v*); 14–15 min, 30–50% B; 15–17 min, 50%; 17–18 min, 50–6% B; 18–21 min, 6% B (equilibration). The flow rate was maintained at 1.4 mL/min, the column temperature was held at 25 °C, and the injection volume was 5 µL. Twelve external standards were employed for calibration, including 5-*O*-caffeoylquinic acid (CHA), 3-*O*- and 4-*O*-caffeoylquinic acids (NCHA and CCHA), caffeoylmalic acid (CAD), (−)-epicatechin (ECA), procyanidins B-2 and C-1 (PB2 and PC1), hyperoside (HP), isoquercitrin (IQ), quercitrin (QR), and rutin (RT). The external standards were also used for quantifying phenolic compounds tentatively identified based on their PDA and MS spectra. Such analytes were quantified as equivalents of the corresponding standards: the levels of proanthocyanidins were calculated as (−)-epicatechin, caffeoylquinic acids as chlorogenic acid, *p*-coumaroylquinic acids as caffeic acid, flavonoid diglycosides as rutin, and flavonoid monoglycosides as hyperoside.

The total phenolic content (TPC) was determined by the Folin–Ciocalteu method [5], and expressed in gallic acid equivalents (GAE). The total proanthocyanidin content (TPA) was analyzed by the n-butanol/HCl method [5] and expressed in cyanidin equivalents (CYE). The tannin content (TTC) was determined by the hide powder test [32] and expressed in pyrogallol equivalents (PRE).

### 4.4. Antioxidant Activity

The antioxidant activity of the plant materials was evaluated in vitro by spectrophotometric methods described previously for DPPH free radical scavenging [5], O_2_^•−^ scavenging in the xanthin/xanthine oxidase system with nitrotetrazolium blue chloride (NBT) used for detection [33], and ferric reducing antioxidant power (FRAP) [5]. The results of the scavenging tests were calculated from concentration–inhibition curves and expressed as normalized effective concentration (EC_50_) values (µg/mL). The FRAP results were calculated from the calibration curve of ferrous sulfate and expressed in mmol of ferrous ions (Fe^2+^) produced by 1 g of the plant materials. The standards of ascorbic acid (AA) and Trolox (TX) were used as positive controls.

### 4.5. Inhibition of Hyaluronidase and α-Glucosidase

The ability of the plant materials to inhibit hyaluronidase and α-glucosidase enzymes was evaluated according to the methods published previously [10,31]. The results were expressed as inhibitory concentration (IC_50_) values (µg/mL) from the concentration–inhibition curves. As the commercial inhibitors, acarbose (AR) and heparin (HP) were used as positive controls.

### 4.6. Statistical and Data Analysis

All data reported in the tables and figures are the mean values ± standard error (SE) of three independent experiments with three replications each. The statistical differences between samples and positive controls were assessed using an ANOVA test, followed by the post hoc Tukey’s test for multiple comparisons. A level of *p* < 0.05 was accepted as statistically significant. The statistical analyses were performed by Statistica 13 Pl software for Windows (StatSoft Inc., Krakow, Poland).

## 5. Conclusions

Given that the highest antioxidant, antihyperglycemic, and anti-inflammatory activities, along with the highest polyphenol levels, were observed in *C. zabelii* leaf samples collected during summer and autumn months (June–July, September–October), these periods appear optimal for harvesting the plant material in Polish climatic conditions. Considering the moderate seasonal variability of both polyphenolic profile and activity parameters, the leaves of *C. zabelii* harvested during the optimized harvest periods appear profitable for industrial plant production and food, are phytotherapeutic, and have cosmetic applications. The biological effects of the *C. zabelii* leaves are mainly driven by the synergistically acting proanthocyanidins, caffeic acid derivatives, and flavonoids, with a primary impact resulting from proanthocyanidins. This study shows the high predictive value of the phytochemical profiles of *C. zabelii* leaves toward their biological potential in oxidative stress, inflammation, and diabetes-related disorders and provides an effective analytical tool for the pre-harvest and post-harvest quality control of the plant material.

## Figures and Tables

**Table 1:** Variability coefficients (CVs) of compound group concentrations across foliar development stages in *C. zabelii.*

Compound Group	CV (%)	Compound Group	CV (%)
TPC (mg/g)	8.29	TFC (mg/g) HPLC	56.76
TPA (mg/g)	7.94	TCFA (mg/g) HPLC	27.56
TTC (mg/g)	19.40	CPCA (mg/g) HPLC	13.41
TLPA (mg/g) HPLC	18.31	TPHA (mg/g) HPLC	26.87

The concentration parameters are according to Figure 2 and Figure 3. Parameters: TPC, total phenolic content; TPA, total proanthocyanidin content; TTC, total tannin content; TLPA, the sum of individual low-molecular weight proanthocyanidins; TFC, the sum of individual flavonoids; TCFA, the sum of individual caffeic acid derivatives; CPCA, the sum of individual *p*-coumaroyloquinic acids; TPHA, the sum of TCFA and CPCA.

**Table 2:** Fluctuations in the antioxidant, antihyperglycemic, and anti-inflammatory activity of *C. zabelii* leaves throughout the growing season from May (V) to October (X).

Sample/Variability	DPPH Scavenging	FRAP	O_2_^•−^ Scavenging	α-Glucosidase Inhibition	Hyaluronidase Inhibition
	EC_50_ (µg/mL) ^a^	mmol Fe^2+^/g ^b^	EC_50_ (µg/mL) ^a^	IC_50_ (µg/mL) ^c^	IC_50_ µg/mL ^c^
TX	4.70 ± 0.09 ^A^	13.54 ± 0.26 ^D^	136.97 ± 1.84 ^E^	-	-
AA	4.13 ± 0.02 ^A^	26.25 ± 0.10 ^E^	3.24 ± 0.11 ^A^	-	-
AR	-	-	-	201.69 ± 2.76 ^D^	-
HP	-	-	-	-	58.93 ± 1.63 ^A^
V	21.59 ± 0.44 ^D^	2.78 ± 0.03 ^A,B^	39.77 ± 1.25 ^D^	44.19 ± 1.39 ^C^	135.87 ± 1.00 ^D^
VI	19.04 ± 0.06 ^B,C^	2.97 ± 0.02 ^B,C^	21.23 ± 0.17 ^B^	36.39 ± 1.46 ^A,B^	120.87 ± 0.48 ^C^
VII	17.97 ± 0.33 ^B^	3.09 ± 0.02 ^C^	19.78 ± 0.48 ^B^	32.62 ± 0.79 ^A^	99.82 ± 0.39 ^B^
VIII	19.06 ± 0.59 ^B,C^	2.73 ± 0.08 ^A^	28.72 ± 1.91 ^C^	44.36 ± 1.07 ^C^	119.88 ± 2.22 ^C^
IX	20.54 ± 0.68 ^C,D^	2.73 ± 0.04 ^A^	32.54 ± 0.21 ^C^	41.10 ± 0.17 ^B,C^	99.88 ± 1.32 ^B^
X	17.27 ± 0.14 ^B^	3.05 ± 0.03 ^C^	30.96 ± 0.51 ^C^	39.29 ± 0.39 ^B^	100.29 ± 1.52 ^B^
CV (%) ^d^	8.31	5.69	25.84	11.56	13.39

Data reported as the mean values ± standard error SE (*n* = 3) based on the dry weight of the plant material harvested monthly (V–X). Reference standards: TX, Trolox; AA, ascorbic acid; AR, acarbose; and HP, heparin. For each test, the different capital letters A–E denote statistically significant differences (*p* < 0.05). ^a^ Radical-scavenging efficiency calculated as EC_50_, effective concentration, the quantity of the plant material or standard required to decrease the initial DPPH or O_2_^•−^ concentration by 50%. ^b^ Results expressed in mmol Fe^2+^ per gram of dry weight of the plant material or standard. ^c^ The ability to inhibit α-glucosidase or hyaluronidase, determined as IC_50_, inhibitory concentration, the amount of an analyte needed to inhibit the enzyme activity by 50%—expressed in micrograms of the dry weight of the plant material or standard per mL of the enzyme solution. ^d^ Variability coefficients (CVs) of biological activity of *C. zabelii* leaves across the growing season (V–X).

**Table 3:** Correlation coefficients (r) and probability values (*p*) of the linear relationships between the phenolic contents of *C. zabelii* leaves and their activity parameters.

*r* (*p*) for:	DPPH-Scavenging EC_50_ (µg/mL)	FRAP mmol Fe^2+^/g	O_2_^•−^-Scavenging EC_50_ (µg/mL)	α-Glucosidase Inhibition IC_50_ (µg/mL)	Hyaluronidase Inhibition IC_50_ (µg/mL)
TPC (mg/g)	−0.9530 (0.003) *	0.9088 (0.012) *	−0.6819 (0.136)	−0.7701 (0.073)	−0.6840 (0.134)
TPA (mg/g)	−0.8346 (0.039) *	0.9553 (0.003) *	−0.7256 (0.103)	−0.9044 (0.013) *	−0.6609 (0.153)
TTC (mg/g)	−0.89016 (0.017) *	0.6813 (0.136)	−0.6011 (0.207)	−0.6458 (0.166)	−0.8869(0.019) *
TLPA (mg/g)	−0.9488 (0.004) *	0.7749 (0.070)	−0.5998 (0.208)	−0.6618 (0.152)	−0.8265 (0.043) *
TFC (mg/g)	−0.6250 (0.185)	0.3640 (0.478)	0.1502 (0.776)	0.0354 (0.947)	−0.4976 (0.315)
TCFA (mg/g)	0.4364 (0.387)	0.0905 (0.865)	0.2065 (0.695)	0.0183 (0.973)	0.7674 (0.045)
CPCA (mg/g)	0.0421 (0.697)	0.1664 (0.753)	0.6048 (0.203)	0.1982 (0.707)	0.3339 (0.518)
TPHA (mg/g)	0.4352 (0.389)	0.0925 (0.862)	0.2151 (0.682)	0.0217 (0.967)	0.7647 (0.047) *

Correlation data for the parameters given in Figure 2 and Table 2. Asterisks denote the statistical significance of the estimated linear relationships (*p* < 0.05).

## Data Availability

Data are contained within the article.

## Abbreviations

UHPLC: Ultra-high performance liquid chromatography; HPLC: high-performance liquid chromatography; PDA: photodiode array; ESI: electrospray ionization; MS: mass spectrometry; DPPH: 2,2-diphenyl-1-picrylhydrazyl; FRAP: ferric reducing antioxidant power, O_2_^−•^: superoxide anion radical.
